# A Label-Free Immunosensor for IgG Based on an Extended-Gate Type Organic Field Effect Transistor

**DOI:** 10.3390/ma7096843

**Published:** 2014-09-22

**Authors:** Tsukuru Minamiki, Tsuyoshi Minami, Ryoji Kurita, Osamu Niwa, Shin-ichi Wakida, Kenjiro Fukuda, Daisuke Kumaki, Shizuo Tokito

**Affiliations:** 1 Research Center for Organic Electronics (ROEL), Graduate School of Science and Engineering, Yamagata University, 4-3-16 Jonan, Yonezawa, Yamagata 992-8510, Japan; E-Mails: tey14898@st.yamagata-u.ac.jp (T.M.); fukuda@yz.yamagata-u.ac.jp (K.F.); d_kumaki@yz.yamagata-u.ac.jp (D.K.); tokito@yz.yamagata-u.ac.jp (S.T.); 2 Biomedical Research Institute, National Institute of Advanced Industrial Science and Technology (AIST), Tsukuba Central 6, 1-1-1 Higashi, Tsukuba, Ibaraki 305-8566, Japan; E-Mails: r.kurita@aist.go.jp (R.K.); niwa.o@aist.go.jp (O.N.); 3 Health Research Institute, National Institute of Advanced Industrial Science and Technology (AIST), 2217-14 Hayashi, Takamatsu, Kagawa 761-0395, Japan; E-Mail: s.wakida@aist.go.jp

**Keywords:** organic field effect transistor, immunosensor, label-free, immunoglobulin G, self-assembled monolayer

## Abstract

A novel biosensor for immunoglobulin G (IgG) detection based on an extended-gate type organic field effect transistor (OFET) has been developed that possesses an anti-IgG antibody on its extended-gate electrode and can be operated below 3 V. The titration results from the target IgG in the presence of a bovine serum albumin interferent, clearly exhibiting a negative shift in the OFET transfer curve with increasing IgG concentration. This is presumed to be due an interaction between target IgG and the immobilized anti-IgG antibody on the extended-gate electrode. As a result, a linear range from 0 to 10 µg/mL was achieved with a relatively low detection limit of 0.62 µg/mL (=4 nM). We believe that these results open up opportunities for applying extended-gate-type OFETs to immunosensing.

## 1. Introduction

Recent efforts in biomedical research have been focused on preventative healthcare and rapid diagnoses of incipient diseases [1]. Immunoassays are one of the most common biochemical tests based on their ability for specific recognition of an antibody to bind a biomolecule and are commonly employed as diagnostic tools [2]. Many types of immunoassays, such as radioimmunoassay (RIAs), enzyme-linked immunosorbent assays (ELISAs), fluorescent and chemiluminescent immunoassays, have been developed, because of their specificity and sensitivity [3]. However, these immunoassays are relatively complicated, due to the necessity of labels; for example, RIAs and ELISAs need radioisotopic and enzymatic labels, respectively. Accordingly, label-free detection of biomolecules based on a quartz crystal microbalance (QCM) [4] or surface plasmon resonance (SPR) [5] have also been developed. In particular, immunosensors based on field-effect-transistors (FETs) are simpler and can be integrated readily into compact sensor devices [6].

Furthermore, organic FETs (OFETs) can potentially be applied to disposable immunosensors, because of their low fabrication costs. Therefore, we believe that the OFET sensors are one of the more promising alternatives as a platform for emerging healthcare devices [7]. Very recently, we succeeded in developing an extended-gate-type OFET, within which the extended gate surface is functionalized with streptavidin for the specific detection of biotinylated-immunoglobulin G (IgG) [8]. The operation mechanism of extended-gate-type FET sensors can be explained by an interfacial potential shift at the gate/solution interface. The charge of captured proteins affects the channel conductance, and subsequently, the threshold voltage is shifted [9,10,11]. The success of this research work allows us to propose a new extended-gate-type OFET immunosensor for IgG detection. Although the detection of biotinylated IgG was successfully achieved, more practical biosensors should be performed without biotin labeling of antibodies. In this paper, we report on the immunosensing of unlabeled IgG (=target IgG) in an aqueous solution using an extended-gate-type OFET.

## 2. Results and Discussion

### 2.1. Design of the OFET Immunosensor Device and Functionalization of the Extended Gate

We designed an immunosensor using an extended-gate-type OFET, based on research work that assumes that IgG detection is performed in water. It is known that the concentration of IgG is related to connective tissue diseases [12]. In the device, the organic transistor portion of the sensor is separated from the detection site, thus water-induced degradation of the organic transistor can be prevented [13]. A gold (Au) layer on a plastic film was employed as the extended-gate electrode, allowing us to functionalize its surface for the immunosensing process. We decided to immobilize streptavidin [14], which is capable of binding biotinylated anti-IgG antibody, whereby the detection of the corresponding IgG can be performed.

**Figure 1:**
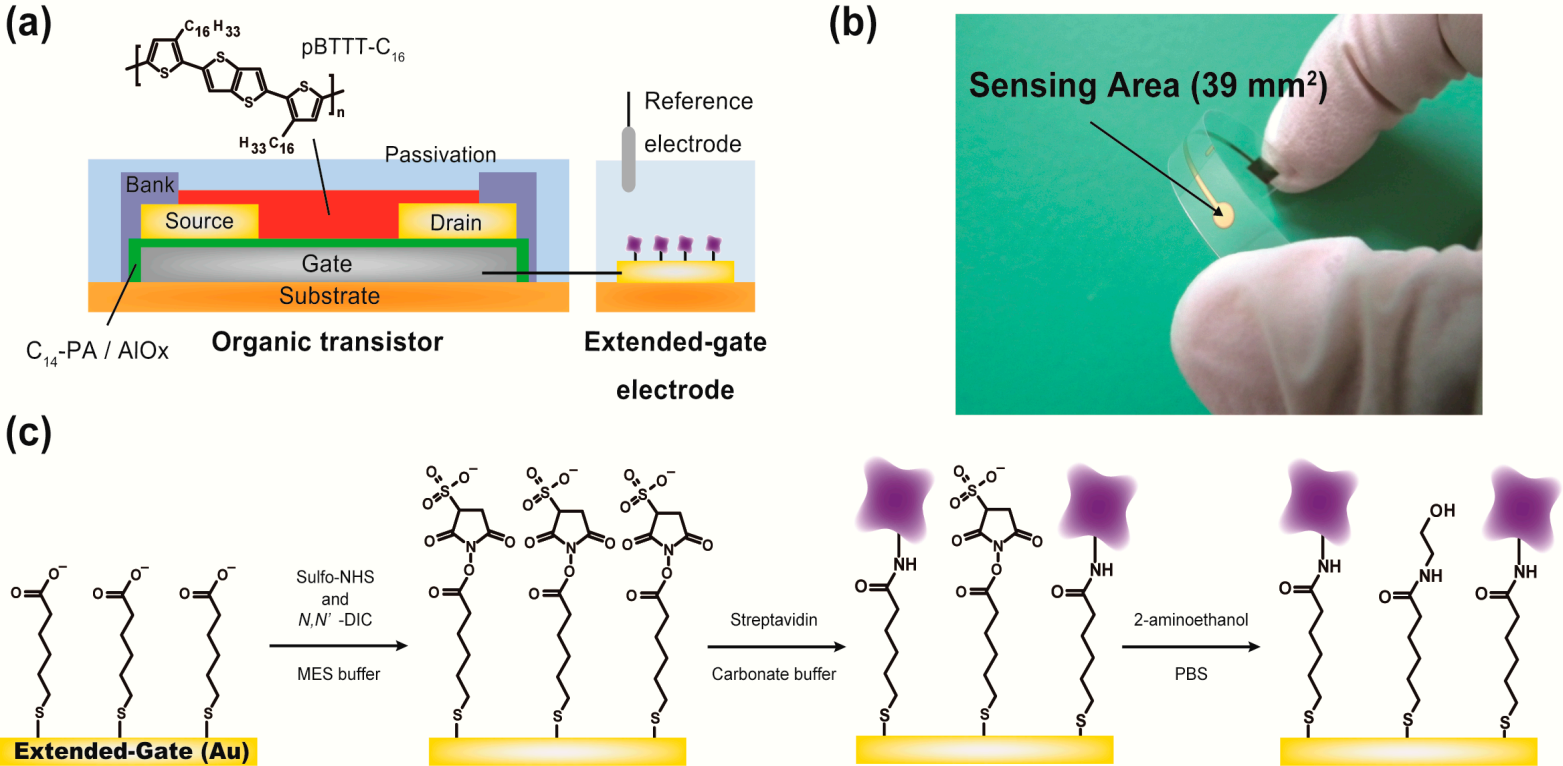
(**a**) Schematic structure of the extended-gate-type organic transistor; (**b**) a photograph of the sensing part (*i.e.*, the extended-gate electrode); and (**c**) the reaction scheme for the immobilization of streptavidin on the surface of the extended-gate electrode. Unreacted *N*-hydroxysulfosuccinimide (sulfo-NHS) esters are treated with 2-aminoethanol.

The structure for the extended-gate-type OFET device is illustrated in Figure 1a. The transistor was especially designed for low-voltage operation. The details of the device fabrication are described in the Experimental Section. It is important to note that we avoided using photolithography processes to simplify fabrication, which is a significant difference from our recent report [8].

**Figure 2:**
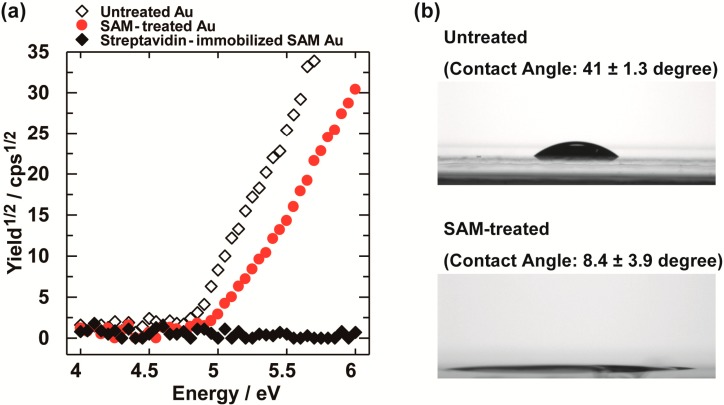
(**a**) Photoelectron yield spectroscopy measurements of the Au electrodes in air. Untreated Au (white diamond), 5-carboxy-1-pentanethiol-treated Au (red circle) and streptavidin-immobilized self-assembled monolayer (SAM) Au (black diamond); and (**b**) water contact angle measurements on the Au electrodes before and after SAM treatment.

We investigated the formation of 5-carboxy-1-pentanethiol self-assembled monolayer (SAM) and the immobilization of streptavidin on the Au electrode with photoelectron yield spectroscopy in air. Photoelectron yield spectroscopy measurements showed a higher ionization potential on the 5-carboxy-1-pentanethiol-treated Au electrode (5.03 ± 0.09 eV) than an untreated Au electrode (4.80 ± 0.01 eV) (Figure 2a), indicating that the electronegative functional group covered the Au surface [15].

Moreover, we measured photoelectron yield spectroscopy after the incubation of streptavidin. As a result, the measurement did not show photoelectric effects (Figure 2a, black diamond). This is presumed to be due to the prevention of photoelectric effects by fully covering the Au electrode with streptavidin and is in good agreement with our previous report [8].

In addition, we measured a water contact angle on the SAM-treated Au electrode using a contact angle goniometer (Figure 2b). The water contact angle of the 5-carboxy-1-pentanethiol-treated Au electrode (~8°) was much lower than that of the untreated Au electrode (41°). Recently, we reported a contact angle of a 10-carboxy-1-decanethiol-treated SAM Au electrode that was slightly higher than that of the untreated Au electrode [8]. An explanation for these differences is thought to be the length of the alkyl chain. Because the hydrophilicity of the 5-carboxy-1-pentanethiol-treated SAM Au electrode was very high, we could not observe a significant difference in the wettability of the Au electrodes before and after the incubation of streptavidin.

### 2.2. Immobilization of Anti-IgG Antibody

The electrical characteristics of the OFET device were measured. As expected, the fabricated OFET device functioned reproducibly below 3 V, suggesting that the device is suitable for immunoassay applications. To fabricate the immunosensor for target IgG, we immobilized biotinylated anti-IgG antibody on the extended-gate electrode of the OFET. A Dulbecco’s phosphate buffer saline (D-PBS) solution of a biotinylated anti-IgG antibody with 0.1 wt% bovine serum albumin (BSA) was cast onto the extended-gate electrode, followed by incubation for 30 min at room temperature.

**Figure 3:**
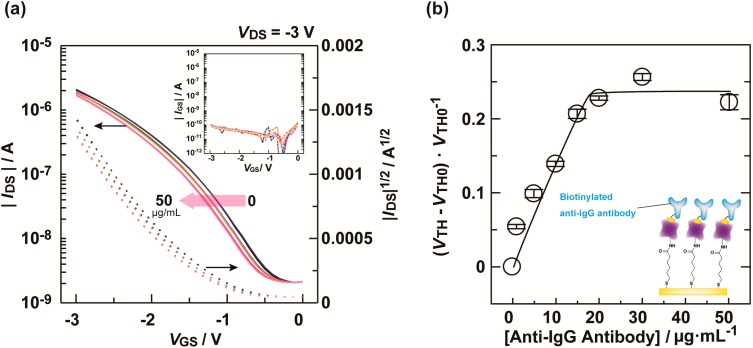
(**a**) Transfer characteristics (*I*_DS_*–V*_GS_) of the OFET upon titration with biotinylated anti-IgG antibody in a D-PBS solution with 0.1 wt% BSA. [Anti-IgG antibody] = 0–50 μg/mL. The inset shows the gate-source current (*I*_GS_–*V*_GS_); (**b**) Changes in threshold voltage (*V*_TH_) for the OFET by biotinylated anti-IgG antibody at various concentrations in a D-PBS solution with 0.1 wt% BSA.

Figure 3a shows the transfer characteristics of the OFET upon titration with the D-PBS solution of biotinylated anti-IgG antibody. The titration results showed a negative shift of the transfer curve with increasing of the biotinylated anti-IgG antibody concentration. This shift is attributed to carrier concentration changes in the OFET channel by the anti-IgG antibody captured on the extended-gate electrode [16]. Importantly, no significant changes in the gate current were observed, meaning that an electrochemical reaction did not occur at the gate [10,17]. Figure 3b shows the relationship between the anti-IgG antibody concentration changes in the threshold voltage. According to the titration results, we decided to use 30 μg/mL biotinylated anti-IgG antibody for complexation of streptavidin with the biotin moiety.

### 2.3. Label-Free Immunoassay for IgG

**Figure 4:**
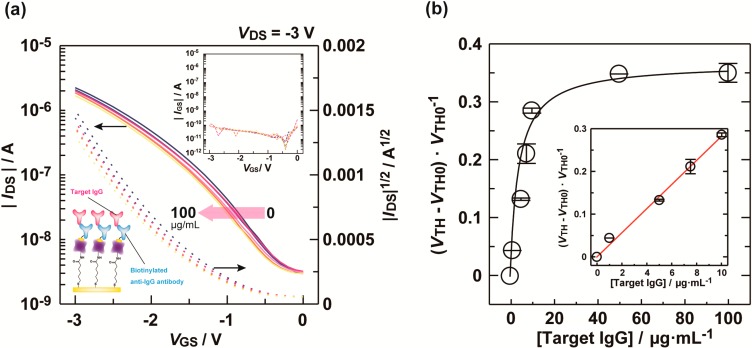
(**a**) Transfer characteristics (*I*_DS_*–V*_GS_) of the OFET upon titration with target IgG in a D-PBS solution with 0.1 wt% BSA. [Target IgG] = 0–100 μg/mL. The inset shows the gate-source current (*I*_GS_*–V*_GS_); (**b**) Changes in threshold voltage (*V*_TH_) for the OFET by target IgG at various concentrations in a D-PBS solution with 0.1 wt% BSA. The inset shows the lower end of the titration.

Finally, we attempted to achieve an immunoassay for target IgG using the extended-gate OFET modified with the anti-IgG antibody. The antibody modified extended-gate was incubated in a D-PBS solution of the target IgG with 0.1 wt% BSA for 1 h at 37 °C. After this period, we measured the transfer characteristics of the OFET. For each analyte, five repetitions were measured using the same OFET device. The titration results of the target IgG are summarized in Figure 4. A negative shift of the transfer curve with increasing of the target IgG concentration was observed, indicating that our designed immunosensor based on the extended-gate OFET performed sufficiently well in the presence of a large excess of BSA interferent. The response of the OFET biosensor is not influenced by the BSA interferent [8]. This suggests that the sensor device might be used for IgG quantification in a complex medium, such as plasma or milk. Although the extended gate is not recyclable, the fabricated OFET part is reusable.

We obtained a linear relationship in the region of the low concentration (0–10 µg/mL). The limit of detection (LOD) [18] and the limit of quantification (LOQ) [18] were estimated to be 0.62 µg/mL (=4 nM) and 2.1 µg/mL (=15 nM), respectively. It is worthwhile to note that the obtained LOD and LOQ values were estimated under the condition of the existence of the BSA interferent. The sensitivity of the fabricated OFET sensor is higher than that of some reported OFETs for protein sensing [19]. In addition, the obtained sensitivity is comparable to or higher than other electrochemical approaches reported in the literature [20,21].

## 3. Experimental Section

### 3.1. Materials and Equipment

Reagents and solvents used for this study were commercially available and used as supplied. D-PBS and octadecylphosphonic acid were purchased from Sigma-Aldrich Inc. (St. Louis, MO, USA). Cytop^®^ (CTL-809M) was purchased from Asahi Glass Co. Ltd. (Tokyo, Japan). Polyethylene naphthalate (PEN) film was purchased from Teijin DuPont Films (Tokyo, Japan). Poly(2,5-bis(3-hexadecylthiophene-2-yl)thieno[3,2-*b*]thiophene (pBTTT-C_16_) [22,23] was purchased from Merck KGaA (Darmstadt, Germany). Gold and aluminum were purchased from Tanaka Kikinzoku Kogyo (Tokyo, Japan) and Furuuchi Chemical Co. (Tokyo, Japan), respectively. FC-43 fluorinert and Teflon^®^ AF1600 were purchased from 3M Co. (St. Paul, MN, USA) and Dupont (Wilmington, DE, USA), respectively. 2-aminoethanol was purchased from Tokyo Kasei (Tokyo, Japan). *N*-hydroxysulfosuccinimide was purchased from Thermo Fisher Scientific Inc. (Waltham, MA, USA). 5-Carboxy-1-pentanethiol and 2-morpholinoethanesulfonic acid were purchased from Dojindo Laboratories (Kumamoto, Japan). Methanol, 2-propanol, hexane, *N,Nʹ*-diisopropylcarbodiimide, sodium chloride and Tween 20 were purchased from Kanto Kagaku (Tokyo, Japan). Streptavidin, 1,2-dichlorobenzene and BSA were purchased from Wako (Osaka, Japan). Mouse IgG (ab37355) and goat anti-mouse IgG H&L (biotin) (ab6788) were purchased from Abcam plc (Cambridgeshire, UK). The aqueous solutions were prepared using Milli-Q water (18 MΩ cm at 25 °C).

Metal electrodes were deposited by using vacuum evaporator equipment from Cryovac, Co. (Osaka, Japan). An oxygen-plasma treatment was performed on PC-300 plasma cleaners from Samco, Inc. (Kyoto, Japan). The UV ozone treatment was by a UV253H UV ozone cleaner from Filgen, Inc. (Aichi, Japan). The bank layers were prepared using IMAGEMASTER 350 dispenser equipment from Musashi Engineering, Inc. (Tokyo, Japan). Photoelectron spectroscopy measurements in air were performed using an AC-3 from Riken Keiki, Co. (Kanagawa, Japan). Wettability measurements were performed on a Theta T200 contact angle goniometer from Biolin Scientific, Co (Stockholm, Sweden). The Ag/AgCl electrode as the reference electrode was purchased from BAS, Inc. (Tokyo, Japan). The incubation was performed using a ICI-100 cool incubator from AS ONE (Osaka, Japan). The electrical characteristics of the all OFET devices were measured using a Keithley 2636B source meter.

### 3.2. Fabrication of the Device

An aluminum (Al) gate electrode was deposited on a glass substrate by thermal evaporation (30 nm in thickness). The gate dielectric consists of a thin-film of aluminum oxide layer (5 nm in thickness) and a tetradecylphosphonic acid (1.7 nm in thickness) SAM [24,25]. The aluminum oxide layer was formed with an oxygen-plasma treatment of the Al gate electrode, whereby the plasma power was 300 W and the treatment duration was 50 min. The SAM was prepared by immersing the substrate in a 2-propanol solution of tetradecylphosphonic acid at room temperature. Au source-drain electrodes (30 nm in thickness) were deposited on the gate dielectric layer using thermal evaporation and patterned using a shadow-mask. The channel width and length of the OFET device were 1000 and 50 μm, respectively. To prepare the bank layers, a 1 wt% solution of an amorphous fluoropolymer in FC-43 was dispensed using the dispenser equipment. Subsequently, a semiconducting polymer, poly(2,5-bis(3-hexadecylthiophene-2-yl)thieno[3,2-*b*]thiophene (pBTTT-C_16_), was drop-casted from a 0.03 wt% solution of 1,2-dichlorobenzene and then annealed at 175 °C for 30 min in a nitrogen atmosphere. To passivate the device, Cytop^®^ (CTL-809M) was spin-coated onto the device and baked at 100 °C for 10 min (100 nm in thickness). Finally, an extended-gate electrode consisting of Au was prepared on a PEN film substrate (125 μm in thickness) using thermal evaporation, such that the sensing area for the extended-gate electrode was 39 mm^2^.

The scheme for immobilizing streptavidin on the extended-gate electrode is summarized in Figure 1c. The Au extended-gate electrode was immersed in a hexane solution containing 1 mM of 5-carboxy-1-pentanethiol for 1 h at room temperature to form the SAM. The treated electrode was washed by ethanol and water, and then 5 μL of a 2-morpholinoethanesulfonic acid (MES) buffer solution (100 mM, pH 6.0) containing *N*-hydroxysulfosuccinimide (sulfo-NHS, 5 mM), *N,Nʹ*-diisopropylcarbodiimide (DIC, 40 mM) and sodium chloride (500 mM) were dropped onto the electrode. The reaction time of the sulfo-NHS ester formation was 15 min.

After this period, streptavidin (500 μg/mL) in 5 μL of a carbonate buffer solution (Na_2_CO_3_: 15 mM, NaHCO_3_: 35 mM, pH 9.6) was applied in drops onto the electrode, followed by incubation for 15 min. To remove unreacted sulfo-NHS esters, 2-aminoethanol (1 M) in 5 μL of D-PBS (KCl: 2.7 mM, NaCl: 136 mM, KH_2_PO_4_: 1.5 mM, Na_2_HPO_4_: 8.1 mM) was dropped onto the electrode and then left standing for 15 min. Finally, the electrode was immersed in D-PBS containing 0.05 wt% Tween 20 and 0.1 wt% BSA for 15 min.

For the detection of the target mouse IgG, the extended-gate modified with streptavidin was firstly immersed in the D-PBS solution of the biotinylated anti-mouse IgG antibody (30 μg/mL) with 0.1 wt% BSA for 30 min at room temperature. After this period, the extended-gate electrode was washed by a D-PBS solution. Next, the extended-gate modified with the streptavidin-biotinylated antibody complex was immersed in the D-PBS solution of the target IgG (0*–*100 μg/mL) with 0.1 wt% BSA for 1 h at 37 °C. After this incubation, the target IgG was electrically detected by the OFET with no further treatment. All measurements were performed using the same OFET.

### 3.3. Estimation of LOD and LOQ

For the determination of the limit of detection (LOD) and the limit of quantitation (LOQ) toward the target IgG, the intersection of the minimum signal (=*Y*) and the regression line obtained from the value of (*V*_TH_ − *V*_TH0_)/*V*_TH0_ in the dynamic range of the titration curve allowed us to estimate the values of the LOD and the LOQ. The value of *Y* is estimated by the following Equation (1):

*Y* = *V*_THavg_ − *k*σ (1)

*V*_THavg_ and σ are the average value and the standard deviation of threshold voltage in the absence of the target IgG, respectively. LOD: *k* = 3, LOQ: *k* = 10.

## 4. Conclusions

In conclusion, we have developed an immunosensor for unlabeled IgG utilizing the extended-gate-type OFET operating at low voltages. The LOD of unlabeled IgG showed that the extended-gate-type OFET can detect nM levels of unlabeled IgG through changes in the transfer curve of the OFET. We believe that the extended-gate-type OFET immunoassay could pave the way to new immunosensors and healthcare applications. Our preliminary results suggest that further modifications of the sensor device and the use of different bioreceptors would yield various sensing systems. Further development of extended-gate-type OFET biosensors is underway in our laboratory.
